# Loss of STOP Protein Impairs Peripheral Olfactory Neurogenesis

**DOI:** 10.1371/journal.pone.0012753

**Published:** 2010-09-15

**Authors:** Karelle Benardais, Basem Kasem, Alice Couegnas, Brigitte Samama, Sebastien Fernandez, Christiane Schaeffer, Maria-Cristina Antal, Didier Job, Annie Schweitzer, Annie Andrieux, Anne Giersch, Astrid Nehlig, Nelly Boehm

**Affiliations:** 1 INSERM U666, Strasbourg, France; 2 Université de Strasbourg, Faculté de Médecine, Institut d'Histologie, Strasbourg, France; 3 Hôpitaux Universitaires de Strasbourg, Strasbourg, France; 4 INSERM U836, Grenoble Institut of Neurosciences, Grenoble, France; iRTSV-GPC, CEA-Grenoble, France; Université Joseph Fourrier, Grenoble, France; University of Queensland, Australia

## Abstract

**Background:**

STOP (Stable Tubulin-Only Polypeptide) null mice show behavioral deficits, impaired synaptic plasticity, decrease in synaptic vesicular pools and disturbances in dopaminergic transmission, and are considered a neurodevelopmental model of schizophrenia. Olfactory neurons highly express STOP protein and are continually generated throughout life. Experimentally-induced loss of olfactory neurons leads to epithelial regeneration within two months, providing a useful model to evaluate the role played by STOP protein in adult olfactory neurogenesis.

**Methodology/Principal Findings:**

Immunocytochemistry and electron microscopy were used to study the structure of the glomerulus in the main olfactory bulb and neurogenesis in the neurosensorial epithelia. In STOP null mice, olfactory neurons showed presynaptic swellings with tubulovesicular profiles and autophagic-like structures. In olfactory and vomeronasal epithelia, there was an increase in neurons turnover, as shown by the increase in number of proliferating, apoptotic and immature cells with no changes in the number of mature neurons. Similar alterations in peripheral olfactory neurogenesis have been previously described in schizophrenia patients. In STOP null mice, regeneration of the olfactory epithelium did not modify these anomalies; moreover, regeneration resulted in abnormal organisation of olfactory terminals within the olfactory glomeruli in STOP null mice.

**Conclusions/Significance:**

In conclusion, STOP protein seems to be involved in the establishment of synapses in the olfactory glomerulus. Our results indicate that the olfactory system of STOP null mice is a well-suited experimental model (1) for the study of the mechanism of action of STOP protein in synaptic function/plasticity and (2) for pathophysiological studies of the mechanisms of altered neuronal connections in schizophrenia.

## Introduction

STOP protein (Stable Tubulin-Only Polypeptide, for a review, see [1]) is a microtubule-associated protein initially isolated from preparations of rat brain cold-stable microtubules. It is a calmodulin-regulated protein able to induce a high degree of microtubule stability in cold-exposed cells [2]. Particularly abundant in neurons, this protein has been shown to be important for normal neurite formation during neuronal differentiation in cultured neurons [3]. STOP null mice show behavioral deficits (disorganized activity, social withdrawal, impaired maternal behavior), hypersensitivity to amphetamine in postpubertal mice, impaired synaptic plasticity, decrease in hippocampal synaptic vesicular pools and disturbances in the dopaminergic, glutamatergic and nicotinic neurotransmissions [4]–[12] and have been proposed as a mouse model to explore the neurodevelopmental and synaptic impairment hypothesis of schizophrenia [4].

Although STOP null mice do not present major brain anomalies, they show subtle modifications of the olfactory system maturation [13]. As adults, they show cognitive deficits using novel object recognition and olfactory discrimination tasks [14]. Since olfactory and vomeronasal pathways highly express STOP transcripts and protein [4], [15], [16], we hypothesized that STOP protein deficiency may lead in adults to synaptic impairment in this pathway. In rodents, there are two subdivisions in the olfactory system: in the main olfactory system, neurosensory cells (olfactory receptor neurons, ORNs) in the olfactory epithelium (OE) send axons to the main olfactory bulb (OB) where they make synapses with the dendrite of mitral/tufted cells in the OB glomeruli; in the accessory olfactory system, axons arising from the neurosensorial cells of the vomeronasal epithelium (VNE), lying in the vomeronasal organ (VNO) make synapses with mitral/tufted cells in the glomeruli of the accessory olfactory bulb (AOB). The olfactory glomerulus represents a useful model system for synapse analysis: its boundaries are sharply delineated; olfactory axons are the unique input; olfactory presynaptic terminals are glutamatergic. The olfactory system is a highly plastic neuronal network. Olfactory and vomeronasal neurosensorial cells constantly renew life long [17]–[21]. There is a constant loss of neurosensorial cells, which die by apoptosis; they are replaced by new neurons arising from progenitors located in the basal compartment of the OE, which consists of two distinct cell types: horizontal basal cells (HBCs) directly attached to the basal lamina and globose basal cells (GBCs) lying immediately above the HBC layer. GBCs are associated with active proliferation and express early neuronal differentiation markers whereas HBCs divide infrequently and express cytokeratin 5 and 14, but not neuronal markers [22], [23]. Immature neurons arising from cell division express GAP 43 and doublecortin; they differentiate to fully mature neurons expressing OMP (Olfactory Marker Protein) [24] and olfactory receptors at the tip of their dendrite, when establishing synapses with the apical dendrite of mitral/tufted cells in the OB. Experimentally-induced loss of olfactory neurons leads to epithelial regeneration within two months, providing a useful model to evaluate the role played by STOP protein in adult olfactory neurogenesis [25].

In the present work, we first asked whether olfactory synapses were morphologically disturbed in the absence of STOP protein, as are hippocampal synapses. Second, do the synaptic modifications impair normal OE and VNE homeostasis? Third, to get insight into STOP protein function in adult ORN biology, we induced ORN regeneration and analysed both peripheral and central levels at two ages, 3 and 10 months.

We show presynaptic anomalies and impaired neurogenesis, some of the impairments recapitulating features observed in schizophrenia patients. Regeneration of the OE did not modify these anomalies in STOP null mice, but moreover induced abnormal organisation of olfactory terminals within the olfactory glomeruli.

Our results indicate that the olfactory system of STOP null mice is a well-suited experimental model (1) for the study of the mechanism of action of STOP protein in synaptic function/plasticity and (2) for pathophysiological studies of the mechanisms of altered neuronal connections in schizophrenia.

## Results

### Loss of STOP protein induces presynaptic anomalies in the olfactory glomerulus

**Figure 1:**
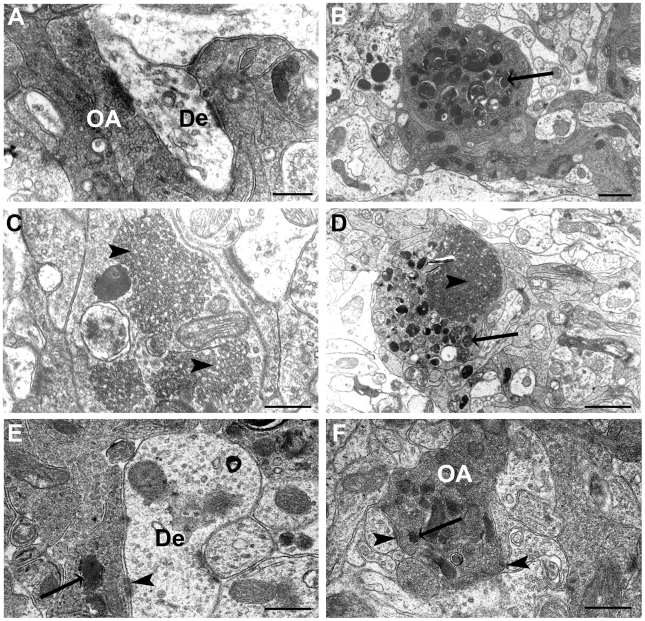
Ultrastructure of olfactory bulb glomeruli. Electron microscopy micrographs of olfactory bulb glomeruli in WT (A) and STOP null (B–F) mice at 3 to 6 months of age. In STOP null mice, olfactory axon endings are filled with autophagic-like structures (B, arrow), tubulovesicular profiles (C, arrowhead), or both (D). When few autophagic structures were present (arrows) (E, F), olfactory axons endings could be identified by the presence of synaptic vesicles and postsynaptic densities (arrowheads) (E, F). De: dentrite; OA: olfactory axon. Scale bar: 0,5 µm (A, C, E, F); 1 µm (B, D).

We first compared synaptic morphology in WT and STOP null mice at 3 to 6 months of age. On semithin sections, no obvious difference could be observed between the two genotypes. At the ultrastructural level however, all STOP null mice displayed the same modifications when compared to WT mice. In glomeruli, olfactory fiber endings are easily recognized by their high electron density (Figure 1A). In both OB and AOB glomeruli of STOP null mice, some axons displayed terminal dilatations containing either autophagic-like structures, (Figure 1B, E, F), complex smooth canalicules (Figure 1C) or both (Figure 1D). These dilatations were recognized as olfactory presynaptic endings by the presence of synaptic vesicles or synaptic densities on the post-synaptic membrane (Figure 1E, F) when few autophagic-like structures were present. They were disseminated in the whole glomeruli layer of the OB and AOB for each animal. They were specific to STOP null mice, since never observed in WT mice (Figure 1A) and different from lipofuscin, which was present in neuronal cell bodies of the oldest mice of the two genotypes. The other layers of the OB and AOB (olfactory fibers, vomeronasal fibers, plexiform, granule and mitral cell layers) did not display any modification in STOP null mice as compared to WT mice.

### Neurogenesis in the olfactory and vomeronasal epithelia

As olfactory and vomeronasal neurons are constantly renewed by peripheral neurogenesis following apoptosis, we asked whether the presynaptic modifications may lead to neurogenesis modifications.

#### Neurogenesis in the OE

The OE was studied at six levels in the rostro-caudal plan. In these six levels, all immunoreactive cells present in the OE of both sides were counted.

**Figure 2:**
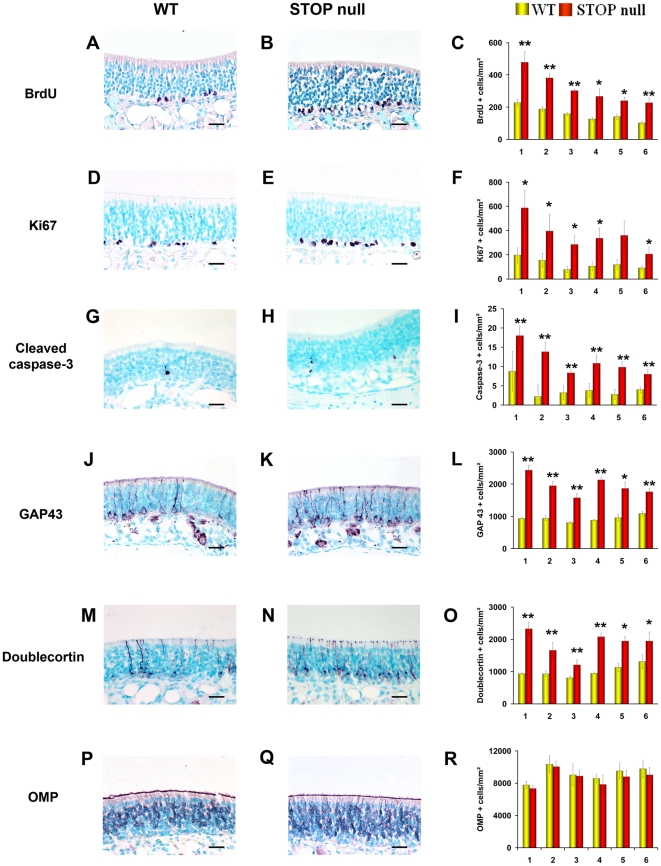
Neurogenesis in the olfactory epithelium. Localisation and mean density of BrdU (A–C), Ki67 (D–F), cleaved caspase 3 (G–I), GAP 43 (J–L), doublecortin (M–O) and OMP (P–R) labelled cells in the olfactory epithelium of WT and STOP null mice. The x-axis refers to the six levels studied, from rostral (level 1) to caudal (level 6). A statistically significant increase in the number of proliferating (BrdU and Ki67 positive cells), apoptotic (caspase 3 positive cells) and immature neurons (GAP 43 and doublecortin positive cells) was observed in STOP null mice as compared to WT mice. There was no difference in the number of mature OMP expressing neurons between the two genotypes. All values are represented as mean +/− SEM, *p<0.05, **p<0.01. Scale bar: 25 µm.

Proliferation was studied by evaluating the immunoexpression of BrdU and Ki67. In the OE of both STOP null and WT mice, positive nuclei were mainly present in the lowest layers, but few superficial cells, either ORNs (round nuclei) or sustentacular cells (elongated nuclei) were also labelled (Figure 2A, B, D, E). There was a statistically significant increase of proliferating cells in STOP null mice as compared to WT mice (Figure 2C, F). The specificity of the increase in proliferation in the sensorial epithelium was shown by the absence of difference in proliferation in the respiratory epithelium between STOP null and WT mice as shown by the mean number of Ki67 positive cells per mm^2^ in the six areas studied for STOP null and WT mice respectively: 4.4±0.5 vs 4.2±0.3; 4.5±0.5 vs 4.7±0.4; 6.4±0.6 vs 5.08±0.7; 14.7±1.2 vs 14.0±1.3; 18.3±0.5 vs 17±2.

Apoptosis was evaluated by counting cleaved caspase 3 positive cells (Figure 2G, H). There was a statistically significant increase in the number of cleaved caspase 3 positive cells in all areas of OE studied (Figure 2I) in STOP null mice when compared to WT mice. Immature neurons were studied by their doublecortin and GAP 43 immunoexpressions. They were localised in the lower part of the epithelium. (Figure 2J, K, M, N). The number of doublecortin and GAP 43 immunoreactive cells was statistically greater in STOP null mice than in WT mice (Figure 2L, O).

There was no statistical difference in the number of mature OMP expressing neurons in WT and STOP null mice (Figure 2P–R).

**Figure 3:**
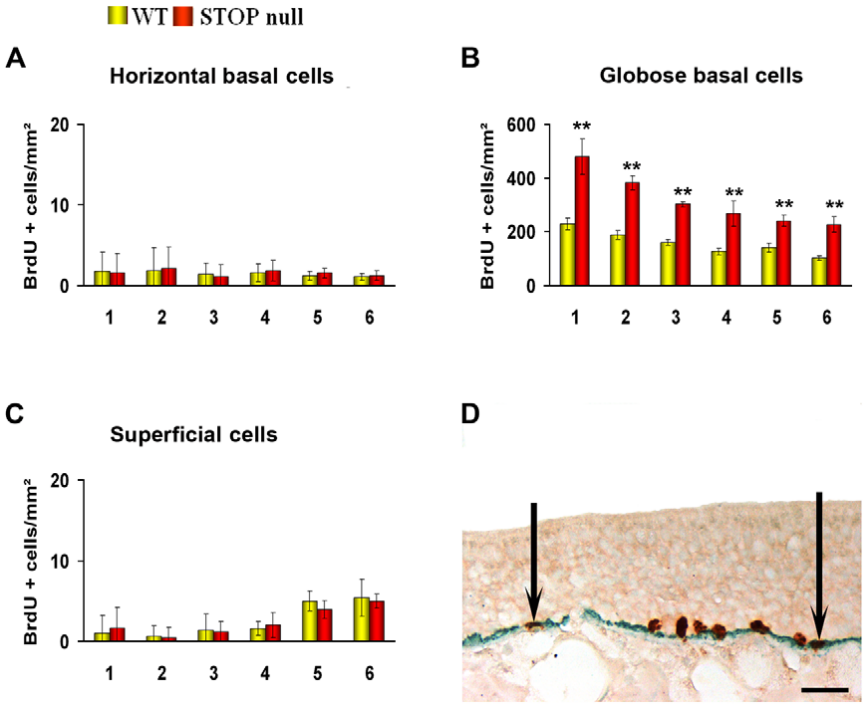
Localisation of BrdU-labelled cells in the olfactory epithelium. Mean density of BrdU-labelled cells in cytokeratine 5 positive HBC layer (A), cytokeratine 5 negative GBC layer (B) and superficial layer (C) in the olfactory epithelium of WT and STOP null mice. The x-axis refers to the six levels studied, from rostral (level 1) to caudal (level 6). Double immunolabelling (arrows) for BrdU (brown) and cytokeratin 5 (green) in the olfactory epithelium of a STOP null mouse is illustrated in D. An increase in globose basal cells but not in horizontal basal and superficial cells was observed in STOP null mice as compared to WT mice. All values are represented as mean +/− SEM, **p<0.01. Scale bar: 30 µm.

We then analysed the distribution of BrdU positive nuclei; cells double-stained for BrdU and cytokeratin 5 were considered as HBCs; nuclei, negative for cytokeratin 5 and localised just above HBC layer were referred as GBCs, and the few remaining superficial cells were considered as superficial cells (Figure 3D). There was an increase in proliferation in GBC layer but not in HBC and superficial cells layers in STOP null mice as compared to WT mice (Figure 3A–C).

#### Neurogenesis in the VNE

Three levels in the rostro-caudal plan of the VNO were studied and all immunoreactive cells present in these levels in the two VNOs of each animal were counted.

**Figure 4:**
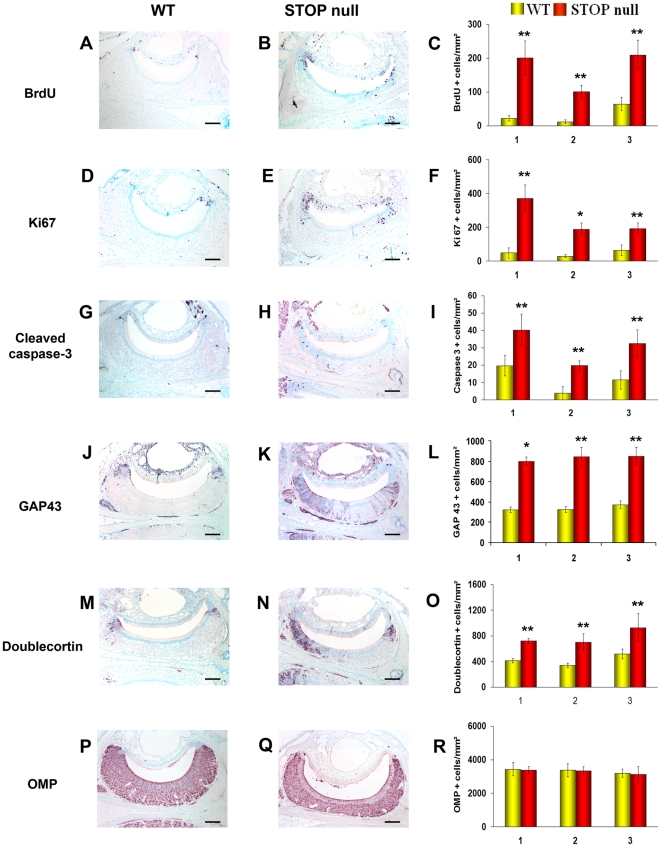
Neurogenesis in the vomeronasal epithelium. Localisation and mean density of BrdU (A–C), Ki67 (D–F), cleaved caspase 3 (G–I), GAP 43 (J–L), doublecortin (M–O) and OMP (P–R) labelled cells in the vomeronasal epithelium of WT and STOP null mice. The x-axis refers to the three levels studied, from rostral (level 1) to caudal (level 3), where the vomeronasal organ was present. A statistically significant increase in proliferating (BrdU and Ki67 positive cells), apoptotic (caspase 3 positive cells) and immature neurons (GAP 43 and doublecortin positive cells), but not mature OMP positive neurons was observed in STOP null mice as compared to WT mice. All values are represented as mean +/− SEM, *p<0.05, **p<0.01. Scale bar: 100 µm.

As for the OE, there was a statistically significant increase in the number of proliferating (Figure 4A–F), apoptotic (Figure 4G–I) and immature cells (Figure 4J–O) but not mature neurons (Figure 4P–R) in STOP null mice as compared to WT mice. In the VNE of both WT and STOP null mice, proliferating and immature cells were mainly but not exclusively located at the margins of the neurosensorial epithelium (Figure 4A–E, J–N). We then asked whether the difference in cell proliferation between the two genotypes resulted from marginal, central or both proliferations. We observed that cells at the margins represented, in WT and STOP null mice, 71%±10 and 63%±8 of the whole proliferating cells, respectively; there was a statistically significant increase in proliferating cells in STOP null mice as compared to WT mice, both for marginal and central localisations (p<0.05).

### Effect of olfactory regeneration on glomerular ultrastructure and peripheral neurogenesis

To gain more insights in the mechanisms of synaptic and neurogenesis disturbances in STOP null mice, we created a complete degeneration of the OE of the right nasal cavity in 3- and 10-month-old animals and analysed two months later epithelial regeneration and glomerular structure. ZnSO4 infusion in the right naris resulted in complete detachment of the OE 48 hours later, without impairing the VNE.

#### Epithelial regeneration and neurogenesis

**Figure 5:**
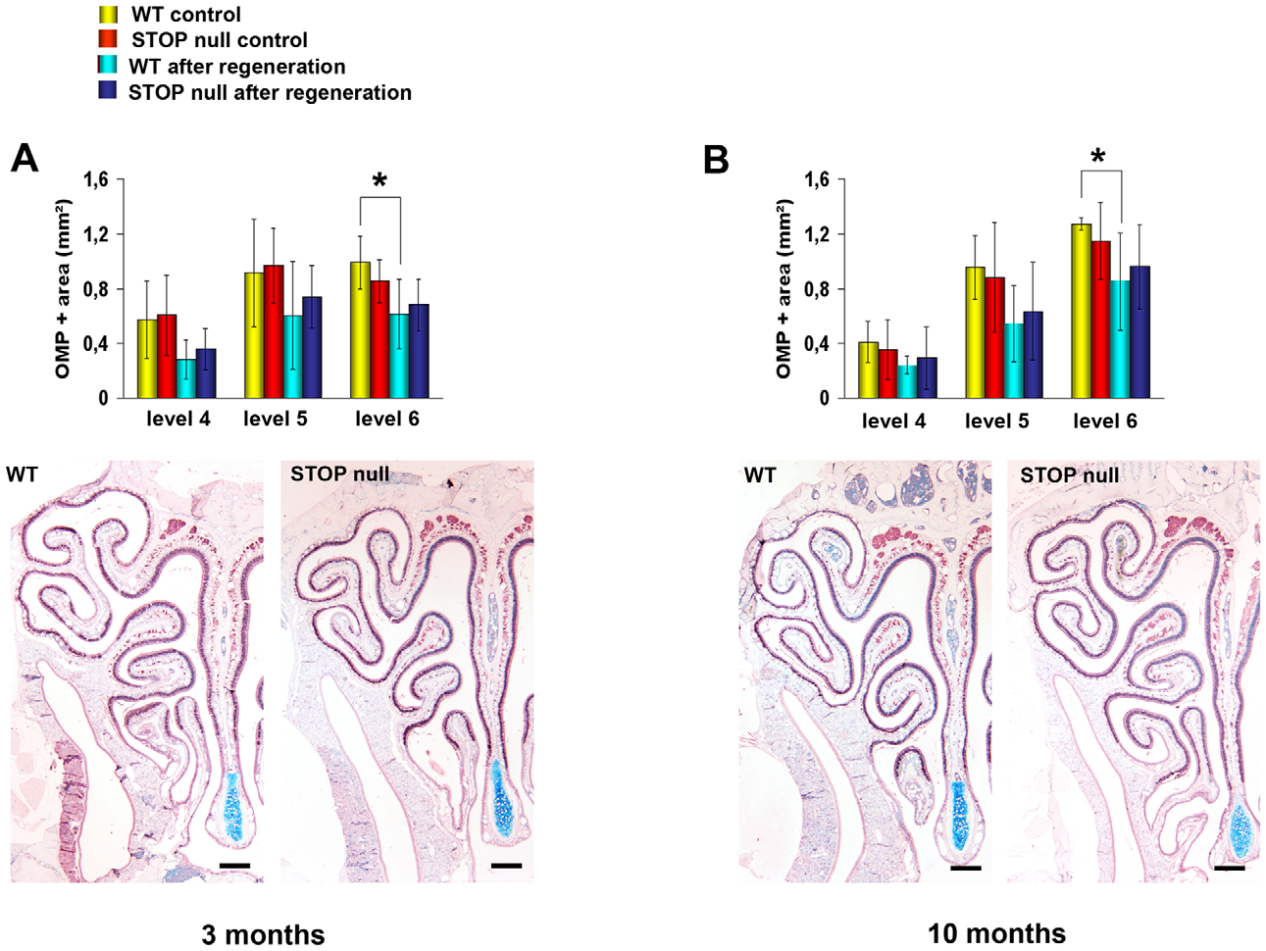
Regeneration of the olfactory epithelium. Mean epithelial OMP positive area before and after regeneration at the three levels 4, 5, 6 in WT and STOP null mice at two different ages. The x-axis refers to the three levels studied, from rostral (level 4) to caudal (level 6), where turbinates are most developed and olfactory epithelium most abundant. There is no difference in the ability of olfactory epithelium to regenerate at the three levels studied between WT and STOP null mice in 3 month-old (A) and 10 month-old (B) animals. All values are represented as mean +/− SEM, *p<0.05. The photomicrographs in A and B illustrate OMP immunostaining in the olfactory epithelium of animals after regeneration. Scale bar: 500 µm.

We first evaluated the extent of OE regeneration at three levels of the nasal cavity, where turbinates are most developed and OE most abundant, and observed that there was no difference in the ability of the OE to regenerate in the three levels, between WT and STOP null mice, either for the 3 month-old (Fig. 5A) or the 10 month-old animals (Fig. 5B). This result lead us to consider the mean values between these three levels for the analysis of neurogenesis after regeneration.

**Figure 6:**
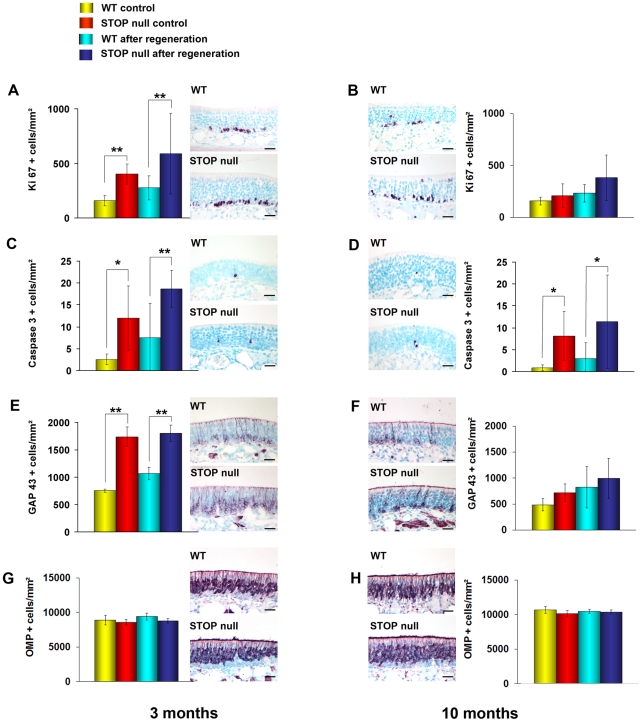
Neurogenesis in the olfactory epithelium after regeneration. Mean density of Ki 67 (A, B), caspase 3 (C, D), GAP 43 (E, F) and OMP (G, H) positive cells in the olfactory epithelium of WT and STOP null mice at two different ages. In the 3-month-old groups (A, C, E, G), apoptotic, proliferating and immature, but not mature neurons are more numerous in STOP null mice as compared to WT mice, both in control animals and after regeneration. In the 10-month-old groups (B, D, F, H) only the number of caspase 3 positive neurons (D) was increased in STOP null mice as compared to WT mice, in controls and after regeneration. All values are represented as mean +/− SEM, *p<0.05, **p<0.01. The photomicrographs illustrate immunostaining in animals after regeneration. Scale bar: 25 µm.

In the 3-month-old groups, apoptotic, proliferating and immature cells were more numerous in STOP null mice as compared to WT mice in both control animals and after regeneration, suggesting no effect of the regeneration itself (Fig. 6A, C, E). The number of OMP positive cells did not differ among the four groups (Fig. 6G). In the 10-month-old groups, similar results were observed for apoptotic (Fig. 6D) and OMP positive cells (Fig. 6H); however, there were no differences between WT and STOP null mice either in controls or after regeneration concerning proliferating (Fig. 6B) and immature cells (Fig. 6F).

We then analysed the OB to check the ability of olfactory neurons axons to target the glomeruli in the absence of STOP protein.

#### Glomerular structure following regeneration

**Figure 7:**
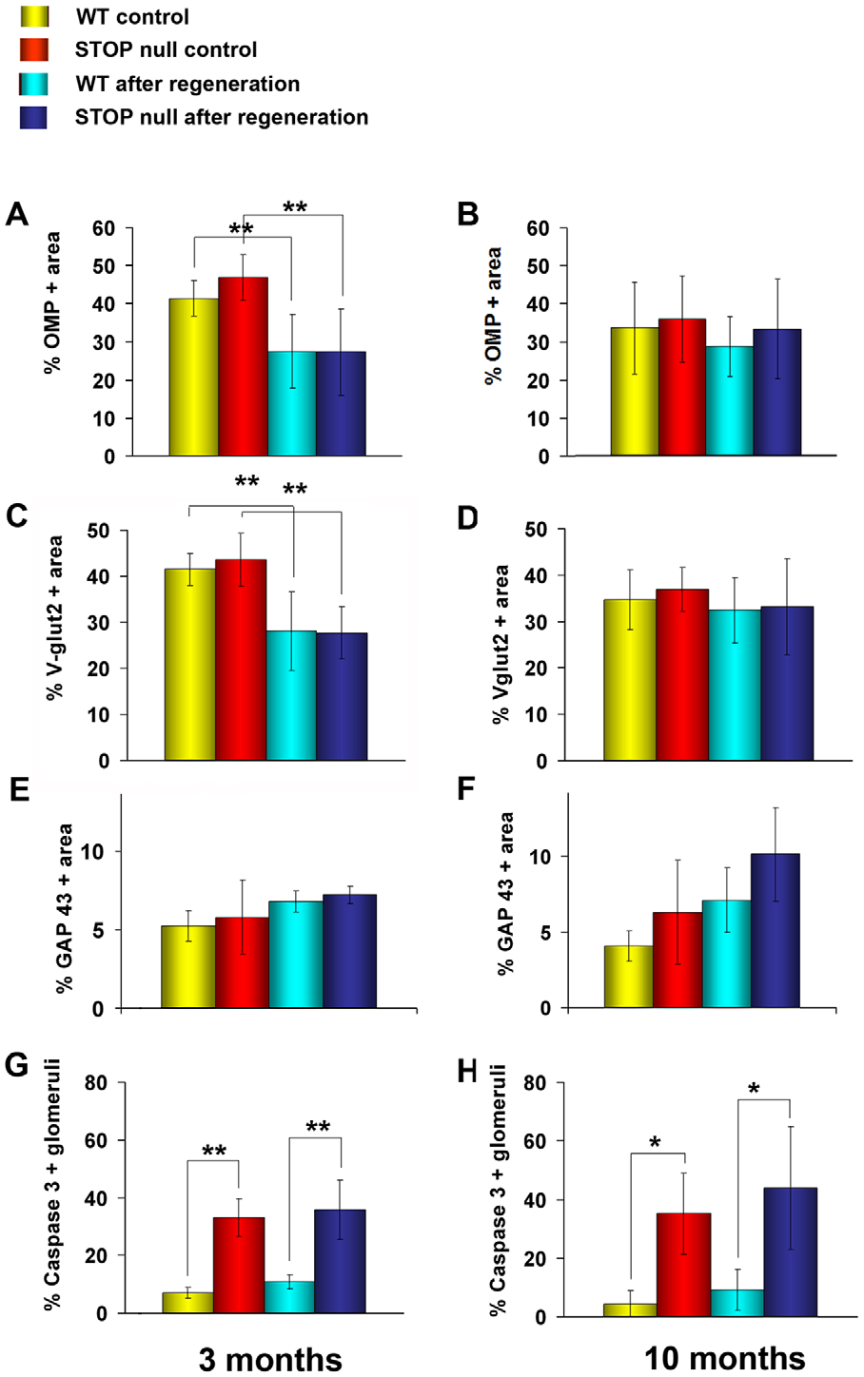
Formation of glomeruli after regeneration. Mean percentage of OMP (A, B), Vglut2 (C, D), GAP 43 (E, F) positive glomerular areas and mean percentage of caspase 3 (G, H) positive glomeruli in WT and STOP null mice at two different ages. In control groups and after regeneration there was no difference between the two genotypes concerning OMP, Vglut2 and GAP 43 immunolabelling at both ages. In controls and after regeneration, the number of glomeruli with apoptotic fibers was greater in STOP null mice as compared to WT mice at both ages. All values are represented as mean +/− SEM, *p<0.05, **p<0.01. The photomicrographs illustrate immunostaining in animals after regeneration. Scale bar: 25 µm.

In control groups and following regeneration, there was no difference between WT and STOP null mice concerning OMP positive fibers (Fig. 7A, B) or olfactory presynaptic Vglut2 densities (Fig. 7C, D) in the glomeruli of 3- and 10-month-old mice. We did not observe statistical differences concerning immature GAP 43 positive fibers between STOP null mice as compared to WT mice (Figure 7E, F). In controls as following regeneration, the number of glomeruli with apoptotic fibers was greater in STOP null mice as compared to WT mice, in 3- and 10-month-old mice (Fig. 7G, H).

**Figure 8:**
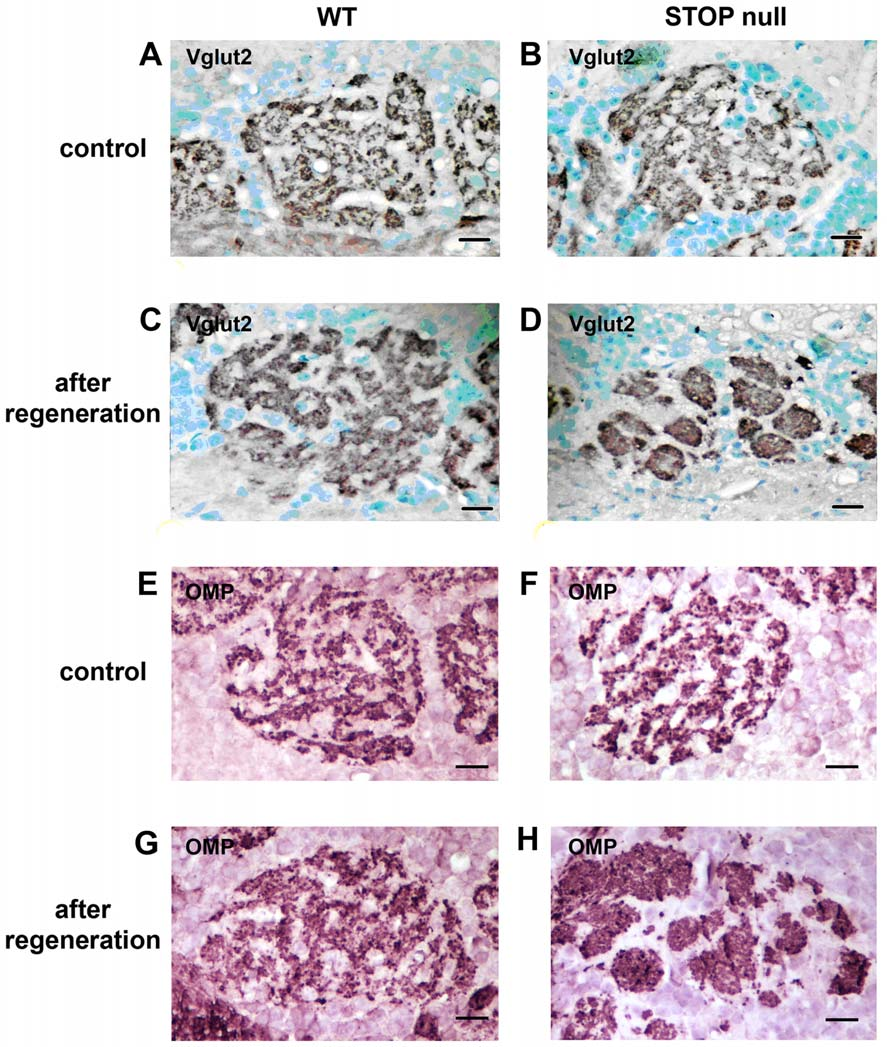
Glomerular structure after regeneration in 10-month-old mice. Localisation of Vglut2 (A–D) and OMP (E–H) immunolabelling in olfactory bulb glomeruli of WT (A, C, E, G) and STOP null (B, D, F H) mice in controls (A, B, E, F) and after regeneration (C, D, G, H). Note the characteristic feature of a mosaicism between Vglut2 or OMP positive fibers and Vglut2 or OMP negative dentrites in WT mice either in controls or after regeneration (A, E and C, G respectively). In STOP null mice, a clumped aspect of either Vglut2 (D) or OMP (H) positive fibers was observed. Scale bar: 20 µm.

Although no quantitative difference could be detected concerning the density of OMP fibers in the glomeruli, we observed however a subtle modification in the distribution of OMP positive fibers and Vglut2 positive terminals in STOP null mice following regeneration: the glomeruli displayed the characteristic feature of a mosaïcism between OMP or Vglut2 positive fibers and OMP negative dendrites in WT mice, either in controls or after regeneration (Figure 8A, C, E, G); in STOP null mice, after regeneration, there was a clumped aspect of OMP or Vglut2 positive fibers in some glomeruli and this feature seemed most obvious in 10-month-old mice (Figure 8B, D, F, H).

**Figure 9:**
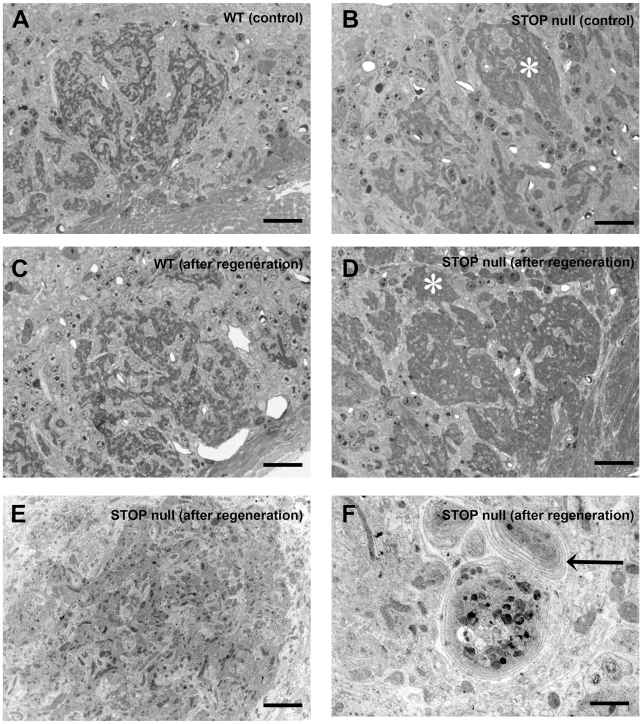
Glomeruli ultrastructure after regeneration in 10-month-old mice. Semithin sections (A–D) and electron microscopy micrographs (E, F) of olfactory bulb glomeruli in WT (A, C) and STOP null (B, D, E, F) mice. In STOP null controls, some olfactory endings are densely packed (*) (B); after regeneration this aspect is more pronounced (D). E illustrates the ultrastructure of a densely packed terminal area in D (*); olfactory axons are densely packed and associated with few electron clear dendrites area. F illustrates concentrically organized axon terminals (F, arrow) in STOP null mice following regeneration. Scale bar: 20 µm (A–D); 5 µm (E); 3 µm (F).

We then analysed the effect of regeneration on glomeruli ultrastructure in 10-month-old mice. On semithin sections, WT mice displayed the characteristic mosaïcism between dense olfactory terminals and clear dendrites, in controls (Figure 9A) and following regeneration (Figure 9C). In STOP null mice, controls displayed most often the same pattern, but a few glomeruli appeared with more condensed olfactory terminals (Fig. 9B). This feature was exaggerated following regeneration (Fig. 9D). At the ultrastructural level, regeneration did not modify the distribution of olfactory terminals and mitral and periglomerular dendrites in WT mice. In STOP null mice, we found the same olfactory terminals anomalies previously described, both in controls and after regeneration: presynaptic dilatations containing most often autophagic-like structures were present, but their number was not increased following regeneration. Ultrastructural analysis confirmed the anomaly of olfactory fibers distribution (Fig. 9E): olfactory terminals were densely packed, in contact with few dendrites; in some glomeruli, concentrically organized axonal terminals profiles were present (Fig. 9F).

## Discussion

In the present work, we show that the absence of STOP protein in the neurosensorial olfactory and vomeronasal cells leads to presynaptic abnormalities in the OB glomeruli and disturbed neurogenesis in OE and VNE. Regeneration in adulthood does not modify this pattern although glomerular organisation is disturbed in the oldest animals following regeneration.

In the olfactory glomeruli of STOP null mice, a very striking result was the presence of olfactory terminal swellings within the olfactory glomeruli with autophagic-like and/or tubulo-vesicular structures. It has been previously shown that in STOP null mice, there was a decrease in synaptic vesicles of the glutamatergic presynaptic endings in CA1 [4]; here, the high electron density within the olfactory endings did not allow us to count with enough accuracy the number of vesicles; so we cannot exclude that this depletion also occurs.

Two hypotheses may explain our results: 1° a disturbance of the anterograde/retrograde transport, in relation with the MAP function of STOP protein [1]–[3]; 2° a disturbance of synaptic vesicles turnover, in relation with the action of the phosphorylated form of STOP protein [26]. The capacity of the STOP null axons to regenerate and project toward the OB glomeruli favors the second hypothesis. The tubulovesicular structures were similar to those observed in nerve terminals of the neuromuscular junction following intoxication with 2,4-dithiobiuret that are associated with a decrease of synaptic vesicles density, suggesting an impairment of vesicle release and recycling [27]. At the presynaptic level, STOP protein is phosphorylated by calmodulin-dependant protein kinase II, an enzyme involved in synaptic plasticity. Phosphorylated STOP protein no longer binds microtubules, but binds actin and colocalizes with synaptic proteins [26]. Indeed, F-actin is strongly aggregated in olfactory glomeruli in many species [28] and the actin cytoskeleton plays a role in the regulation of synaptic vesicle dynamics at the post and pre-synaptic level [29]. Such a mechanism may be in accordance with our results as we only observed olfactory fibers modifications at the most terminal presynaptic level.

Peripheral olfactory neurogenesis was highly disturbed in STOP null mice. It is well known that ORNs are continually replaced throughout life via apoptosis in a caspase-dependent mechanism [30]. In STOP null mice, we observed an increase in the number of cleaved caspase 3 positive neurosensorial cells and fiber endings in the OB glomeruli. Caspase 3 is an effector caspase, which mediates the terminal stages of apoptosis. It has been shown that caspase 3 can be locally activated in synapses, triggering local degeneration without initiating an irreversible apoptotic cascade [31], [32]. However, here we observed activated caspase 3 immunoreactivity in the whole ORN from pericaryon to presynaptic endings, associated with enhanced proliferation, favoring a whole cell apoptotic process. One of the possible cascades leading to apoptosis in the OE has been highlighted using experimental OB deafferentation: caspases are first activated at the site of the lesion, then in the axon and lastly in the cell body in the OE, suggesting a retrograde propagation of cleavage [33]. It is thus tempting to speculate that synaptic signals are triggered by a disturbance of synaptic functioning, leading to exaggerated apoptosis in ORNs. This hypothesis is reinforced by the fact that both peripheral and central differences between the two genotypes are not modified by regeneration. Our ultrastructural observations would be in agreement with this hypothesis since we observed dilatations filled with membranes, a situation that may disturb presynaptic function and induce apoptosis.

ORN apoptosis and neurogenesis are linked, leading to up- or down-regulation of ORNs turnover [19], [34]. We show an increase in proliferation in both OE and VNE, determining an up-regulation of neurosensorial cell turnover. In the OE, neurogenesis occurs vertically, from basal to mature cells layer; in the VNE, however, there are both vertical neurogenesis in the central part of the epithelium, mainly contributing to cell renewal, and marginal neurogenesis, contributing to both neuronal turn-over and neuronal expansion [21], [35]. Both proliferating populations were expended in STOP null mice, in accordance with the fact that both populations of neurons extend projection to the AOB [35], both being thus vulnerable to the absence of STOP in the axon and presynaptic terminals. In mouse OE, the progeny of GBCs undergo progressive terminal differentiation, from immature ORN expressing doublecortin and GAP 43 to mature ORN expressing OMP. Doublecortin is a microtubule-associated protein playing essential roles in cortical neuronal migration and growth of neuronal processes [36]–[38]. GAP 43, also known as B-50 or neuromodulin, is a growth and plasticity associated protein, highly expressed in neuronal growth-cones during synaptogenesis; it is down regulated in many brain regions after synaptogenesis has stopped, but is retained in regions where plasticity is preserved in adulthood. In the adult olfactory system, immature ORNs in the region just above GBCs, olfactory axons and OB glomeruli are GAP 43 positive [39], [40]. We observed an increase in the number of immature, doublecortin and GAP 43 positive neurons in the OE and VNE without statistically significant change in the number of mature neurons in STOP null mice.

Epithelial regeneration did not modify the difference observed in proliferating and immature olfactory cells as well as the concentration of GAP 43 positive fibers in the glomeruli between the two genotypes in the group of three months old mice. This result favors, at that age point, a primary effect of STOP protein loss on presynaptic function, with secondary effects on peripheral neurogenesis.

However, the difference in proliferating and immature cells numbers between the two genotypes was abolished in 10-month-old mice, both in controls and after regeneration, without differences in the number of mature OMP positive neurons. Previously, Farbman et al [41], using naris occlusion as an experimental model to analyse OE dynamics, observed that the total number of mature neurons was not altered by naris occlusion, although the rate of neurogenesis was substantially reduced. We observed that in STOP null mice, glomerular organisation was disturbed, mainly following regeneration, suggesting impairment in axons terminal pathfinding and increased terminal sprouting. Epithelial homeostasis results from a subtle equilibrium integrating both peripheral, epithelial factors and central OB derived signals. Two hypothesis may be suggested, needing further experiments. In the absence of STOP, the modification of olfactory axons projection with age may modify their rate of survival, with immature neurons entering preferentially into apoptosis. A longer survival time may then lead to lesser proliferation. In that respect, recently, Sultan-Styne et al [42], showed that contrary to the classic model where reduction in target (OB) support reduces ORN life span, when OB neurons were selectively destroyed, “sensory population was surprisingly resilient when post-synaptic neurons were depleted”. A second hypothesis may be that absence of STOP protein, in addition to the impairment of mature neurons, may also progressively disturb stem cells and progenitors biology in the OE. In vitro studies of ORN cultures would be helpful for testing these two hypotheses.

STOP null mice have been proposed as a mouse model for the analysis of synaptic dysconnexion in schizophrenia. Our results on peripheral olfactory neurogenesis in these mice have striking similarities with those observed in the OE of schizophrenia patients. Indeed, Féron et al. [43] and McCurdy et al. [44] showed an increase in cell proliferation in schizophrenia patients ORN cultures. Microarray studies on OE have shown an enhanced expression of genes related to proliferation in schizophrenia patients as compared to controls [43]. Arnold et al. [45] observed an increase in GAP 43 positive immature ORNs on histological sections of schizophrenia patients OE.

In conclusion, we show (1) synaptic anomalies in a second brain area in addition to hippocampus in STOP null mice, in accordance with a role of STOP protein in synaptic function/plasticity and (2) disturbed peripheral olfactory neurogenesis paralleling observations in schizophrenia patients. The olfactory pathway represents then a very useful neuronal circuit to test hypothesis concerning (1) the mechanisms of STOP protein functions at the synapse, (2) neuronal connectivity disturbances as pathophysiological mechanisms involved in developmentally induced synaptic connectivity disturbance and altered neurogenesis in schizophrenia, (3) new therapies for proof of concept for future human treatment [46]–[48].

## Materials and Methods

### Animals

STOP null mice and their WT littermates were generated on a mixed BALBc/129 SvPas and on a pure 129 SvPas background as previously reported by Andrieux et al. [4]. All animals used in the study underwent immunohistochemistry for the detection of STOP protein, resulting in no staining in STOP null mice, and genotyping by PCR as described by Andrieux et al. [4]. All mice were kept under standard housing conditions with a 12-hour/12-hour dark-light cycle. The experiments were carried out in accordance with the European Communities Council Directive of 24 November 1986 (86/609/EEC), and the French Department of Agriculture (License N° 67-95). The protocol was approved by the ethical Animal Research Committee of Louis Pasteur University (CREMEAS #AL/01/19/10/07).

In a first experiment, we searched for differences in glomerular ultrastructure and peripheral neurogenesis between WT and STOP null mice: a first group of 24 mice, 3- to 6- month-old, was used for ultrastructural study of the OBs; a second group of 13 animals was used to analyse proliferation and apoptosis in the OE and VNE on paraffin sections in 3-month-old mice. In a second experiment we analysed, in WT and STOP null mice, the effect of OE regeneration on glomeruli structure and ultrastructure and on peripheral neurogenesis at two age times (3 and 10 months).

### BrdU injection

Animals were given an intraperitoneal injection of the thymidine analogue 5-Bromo-2′deoxyuridine (BrdU; 100 mg/kg body weight, Sigma, Saint-Quentin Fallavier, France; 10 mg/ml diluted in saline solution) as a single dose, 24 hours before sacrifice.

### OE destruction by ZnSO4

OE was destroyed according to the model described by Ducray et al. [49] and Boehm et al. [50] with the following modifications. Animals were anesthetized by i.p. injection of chloral hydrate and local application of xylocaine on the bottom of the right naris. Either ZnSO4 (lesioned animals) or physiological serum (control animals) was injected in the right naris at the dose of 2×10 µl for the 3-month-old animals or 15 µl followed by 10 µl for the 10-month-old animals. The two injections were realized at an interval of 1 minute. Animals were kept under observation during one hour until awakening.

### Tissue preparation for morphological analysis by light microscopy

All animals were anaesthetized with sodium pentobarbital and perfused transcardially with freshly depolymerised 4% paraformaldehyde in 0.1 M phosphate buffer; the head was removed and further fixed for 24 h in the same fixative. Heads were decalcified for 8 days (3-month-old mice) or 15 days (10-month-old mice) in 15% EDTA, embedded in paraffin and 5 µm frontal sections were cut. Every first section at 200 µm distance in the rostro-caudal plan was stained with hematoxylin and eosin (H–E) in order to standardize the levels to study. In the first experiment, six consecutive levels (1 to 6), at 600 µm were analysed; the VNE was present on the first three levels. In the second experiment, where only the OE was of interest, levels 4 to 6 were analysed, level 4 corresponding to the end of the VNO.

### Immunocytochemistry

The following antibodies were used: polyclonal rabbit anti-STOP protein [4], anti-GAP 43 (for the analyses of immature cells in the OE and VNE; 1∶5000, Chemicon, Abcys, Paris, France), anti-cleaved caspase 3 (1∶1000, Cell Signalling, Abcam, Cambrige, UK), anti-doublecortin (1∶5000, Abcam, Cambridge, UK), anti-cytokeratin 5 (1∶5000, Abcam, Cambridge, UK), anti-Vglut2 (Synaptic Systems, Gottingen, Germany), goat polyclonal anti-OMP (1∶5000, Wako Chemicals, Neuss, Germany), mouse monoclonal anti-GAP 43 (for the analysis in the OB; 1∶5000, Sigma, Saint-Quentin Fallavier, France), rat monoclonal anti-BrdU (1∶1000, Abcam, Cambridge, UK), rabbit monoclonal anti-Ki67 (1∶500, Microm Microtech, Francheville, France). Microwave unmasking in citrate buffer (10 mM, pH 6) preceded incubation in the primary antibody. After endogenous peroxidase blocking, secondary biotinylated antibody incubation (1∶200) for 2 h was followed by incubation in avidin-biotin complex (Vectastain Elite kit, Vector Laboratories, Abcys, Paris, France). The peroxidase reaction product was revealed by VIP (Vector Laboratories, Abcys, Paris, France). For BrdU detection, incubation in the primary antibody was preceded by DNA denaturation in 2N HCl. The primary antibody was omitted in negative controls.

Double immunostaining was used to distinguish, in the OE, the HBC (cytokeratin 5 positive) from the GBC (cytokeratin 5 negative) proliferating compartment. BrdU was revealed in a first step using DAB as a chromogen, followed by cytokeratin 5 detection using Histogreen (Vector Laboratories, Abcys, Paris, France) as a chromogen.

### Electron microscopy

Animals were anaesthetized with sodium pentobarbital and perfused with glutaraldehyde (2.5%) for 10 min. The OBs were fixed for 12 additional hours in the same fixative, rinsed in cacodylate buffer (0.1 M, pH 7.4); OBs were sliced, post-fixed in osmium tetroxid and embedded in epon 812. Ultrathin sections were cut on a Leica ultramicrotome and stained with lead citrate and uranyl acetate. Grids were examined on a Siemens transmission electron microscope.

### Quantifications

All slides and grids were blind coded until completion of data analysis. For all cell counts, only stained cell bodies or nuclei were considered as positive. For apoptotic cell count, only cytoplasmic labeling ranging from 5 to 15 µm was considered.

For the study of neurogenesis in the first experiment, all BrdU, Ki67, activated caspase 3, doublecortin, GAP 43 and OMP immunoreactive cells were counted in the OE and VNE of the two naris cavities, at the six levels selected in the rostro-caudal plan. For each animal and each marker, three sections corresponding to each one of these six levels were analysed and a mean number of cells was calculated from these counts. The area covered by the epithelia was measured using ImageJ software (WS Rasband, Image J, US National Institutes of Health, Bethesda, MD, USA; http://rsb.info.nih.gov/ij/). Results were expressed as a mean number of positive nuclei or cells per mm^2^ of epithelium +/− SEM. The areas of the epithelia for each level did not statistically differ between STOP null mice and WT mice (Student's *t*-test). Concerning the VNE, proliferation occurs in two subpopulations of cells, marginal and central cells [51]; we then counted the two populations of Ki67 positive cells in the second of the six levels studied, where the VNO is typically C-shaped. The VNE was divided in angles of 20°, 90°, 160° and 180° (for details see ref 51); marginal and central cells were counted in the most external (0° to 20° and 160° to 180°) and the central (20 to 160°) segments respectively. We calculated the percentage represented by the marginal compartment of proliferating cells and compared for each localisation, marginal and central, the number of proliferating cells between STOP and WT mice.

In the second experiment, following regeneration, only the OE of the right naris, where ZnSO4 was injected, was considered. Epithelium regeneration was analysed by comparing, in WT and STOP null mice, the OMP-positive surface of epithelium lining the right naris at levels 4 to 6. Since there was no difference in the ability of the OE to regenerate at these three levels between WT and STOP null mice (see Results), we considered the mean values between these three levels for the analysis of neurogenesis after regeneration. For each animal and each marker, three sections at levels 4–6 were analysed and a mean value was calculated for each level. For each marker, results are expressed as a mean number of positive cells per mm^2^ of epithelium +/− SEM.

For the studies of glomeruli, the surface labeled by each marker was measured after threshold using ImageJ software. Results are expressed as a percentage of labelled area per glomerular surface.

### Statistical analysis

Statistical differences between the experimental groups were performed using ANOVA, followed by the Scheffé posthoc test. The data are expressed as mean values +/− SEM.
